# Downregulation of aquaporin 3 promotes hyperosmolarity-induced apoptosis of nucleus pulposus cells through PI3K/Akt/mTOR pathway suppression

**DOI:** 10.3389/fgene.2025.1665899

**Published:** 2025-11-05

**Authors:** Yuan Sang, Huiqing Zhao, Jiajun Wu, Ting Zhang, Wenbin Xu, Kaihua Liu, Chang Liu, Ping Li, Yichun Xu, Jianying Zhang, Gang Hou

**Affiliations:** ^1^ Department of Orthopaedics, The Third Affiliated Hospital of Sun Yat-Sen University, Guangzhou, China; ^2^ Department of Orthopaedic Surgery, University of Pittsburgh, Pittsburgh, PA, United States

**Keywords:** intervertebral disc degeneration (IVDD), nucleus pulposus cell (NPC), hyperosmolarity, aquaporin 3 (AQP3), PI3K/Akt/mTOR pathway, reactive oxygen species (ROS)

## Abstract

**Introduction:**

Hyperosmolarity plays a crucial role in the apoptosis of nucleus pulposus cells (NPCs) during intervertebral disc degeneration (IVDD). Aquaporin 3 (AQP3), a membrane channel protein, maintains cellular osmotic balance by facilitating the transport of water and osmolytes. While AQP3 downregulation is associated with disc degeneration, its function in apoptosis under hyperosmotic conditions remains unclear.

**Methods:**

We investigated the effects of hyperosmolarity on AQP3 expression and NPC apoptosis in vitro. Furthermore, AQP3 was overexpressed in NPCs using lentiviral vectors, and its function was pharmacologically inhibited. Key markers of the PI3K/AKT/mTOR signaling pathway, mitochondrial function, reactive oxygen species (ROS) accumulation, and apoptosis were assessed. The therapeutic potential of AQP3 was validated in a rat model of IVDD through histological analysis of disc structure.

**Results:**

In this study, we show that hyperosmolarity leads to a reduction in AQP3 levels, inhibits the PI3K/AKT/mTOR signaling pathway, and triggers mitochondrial dysfunction and the accumulation of ROS in NPCs. Overexpression of AQP3 through lentiviral vectors restores this pathway, mitigates oxidative damage, and decreases apoptosis, thereby preserving disc structure in IVDD rat models. Conversely, pharmacological inhibition of AQP3 worsens extracellular matrix (ECM) catabolism and nucleus pulposus (NP) tissue loss.

**Discussion:**

Our findings indicate that AQP3 deficiency under hyperosmotic conditions contributes to NPC apoptosis by suppressing the PI3K/AKT/mTOR signaling pathway, potentially establishing a pathological cycle of disc degeneration. These results highlight AQP3 as a potential therapeutic target for IVDD.

## Graphical Abstract

**Figure:**
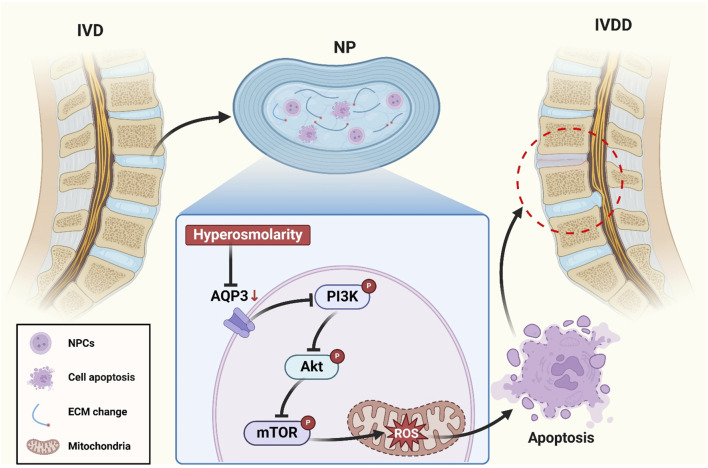
Schematic illustrating the mechanism by which downregulation of AQP3 induces apoptosis in NPCs through suppression of the PI3K/Akt/mTOR pathway in a high osmotic pressure environment.

## Introduction

Lower back pain (LBP), recognized as the primary global cause of disability, predominantly stems from intervertebral disc degeneration (IVDD) - a progressive condition affecting 40% of adults over the age of 40. Beyond its debilitating effect on quality-adjusted life years, IVDD also imposes substantial socioeconomic burdens (GBD, 2017 Disease and Injury Incidence and Prevalence Collaborators, 2018). The multifactorial pathogenesis of IVDD involves a complex interplay of cellular senescence, biomechanical stressors, inflammatory cascades, and genetic predisposition (Wang et al., 2016; Xiang et al., 2024; Zheng et al., 2024; Feng et al., 2016). Despite these mechanistic insights, the precise spatiotemporal regulation of disc degeneration remains poorly understood at the molecular and cellular levels. Current therapeutic paradigms are primarily palliative, offering only temporary relief through physical therapy, pharmacological agents, and invasive surgical procedures (Song et al., 2024; Mroz et al., 2014; Bhadouria and Holguin, 2024; Chou et al., 2007). This critical gap in the availability of disease-modifying therapies highlights an unmet clinical need. Targeted molecular interventions that address the underlying pathobiology of IVDD progression are urgently needed.

The intervertebral disc (IVD) is a sophisticated trilaminar structure comprising the proteoglycan-rich nucleus pulposus (NP), the collagenous annulus fibrosus (AF), and the cartilaginous endplates (Chen et al., 2024). This architectural organization allows the IVD to function as a viscoelastic shock absorber, distributing compressive forces and maintaining spinal flexibility and range of motion (Roberts et al., 2006). The hallmark features of IVDD include progressive cell loss and extracellular matrix (ECM) degradation, particularly within the NP compartment (Vadalà et al., 2019). NPCs reside within one of the most physiologically challenging microenvironments in mammalian systems, characterized by hyperosmolarity, chronic hypoxia, low pH and excessive mechanical stress (Urban and Roberts, 2003). In the IVD microenvironment, the elevated osmotic pressure primarily arises from a proteoglycan-rich ECM. Notably, the physiological osmotic pressure in NP tissue consistently oscillates between 450–550 milliosmoles per kilogram (mOsm/kg), which is significantly higher than that of conventional extracellular fluids in mammalian systems. Increasing evidence suggests that this pathological hyperosmolarity has profound regulatory effects on the homeostasis of NPCs, including metabolic activity, apoptosis resistance, and phenotypic maintenance. However, the mechanisms by which hyperosmolarity regulates NP cell responses remain unclear in IVDD (Li et al., 2016; Li et al., 2017; Ebi et al., 2013). Elucidating these mechanisms could provide critical insights into the pathophysiology of IVDD and reveal novel therapeutic targets.

Local osmotic pressure in the IVD fluctuates markedly. In response, resident cells deploy specific proteins and signaling pathways to adapt osmotically. The aquaporin (AQP) family has emerged as a key regulator of cellular osmoregulation in the IVD microenvironment (Sadowska et al., 2018). AQPs are transmembrane channels that facilitate water transport across lipid bilayers, thereby maintaining cellular osmotic homeostasis (Kozono et al., 2002; Gao et al., 2016; Palacio-Mancheno et al., 2018). In IVDD models, aquaporin expression in embryonic notochordal cells is responsive to osmotic gradients. Transcriptional changes subsequently influence lineage commitment and apoptosis in murine IVD (Richardson et al., 2008). Aquaporin 3 (AQP3), an aquaglyceroporin, exhibits differential expression in the human NP and AF (Richardson et al., 2008; Mobasheri et al., 2004). Proteomic analysis of degenerative discs reveals significant downregulation of AQP3 and impaired glycerol/water transport (Xie et al., 2016). These data support a mechano-osmotic coupling model. AQP3-mediated flux may convert changes in matrix hydration and osmolarity into cytoskeletal remodeling during IVDD (Richardson et al., 2008). Here, we investigate the role of AQP3 in NPC fate under hyperosmotic stress, focusing on the molecular mechanisms driving osmoadaptive reprogramming.

## Materials and methods

### Extraction of rat NPCs

Thirty female Sprague-Dawley rats, weighing 200–220 g and aged 12 weeks, were obtained from Guangdong Sijia Jingda Biotechnology Co., Ltd. (SCXK (Xiang) 2021-0002). The rats were acclimatized for 7 days under specific-pathogen-free (SPF) conditions with unlimited access to autoclaved feed and water. All experimental procedures were performed at Guangzhou Forevergen Biosciences Medical Laboratory Animal Center, following approval by the Institutional Animal Care and Use Committee (SYXK (Yue) 2023-0186).

Euthanasia was performed using gradual CO_2_ displacement. Lumbar intervertebral discs (Co6/7-Co8/9) were aseptically excised within 5 min post-mortem under laminar flow. NP tissue was microdissected from the innermost annular region under a dissecting microscope. Sequential enzymatic digestion was performed in 0.25% trypsin-EDTA (T4049, Sigma-Aldrich, United States) at 37 °C for 5 min with 150 rpm orbital shaking and 0.25% collagenase type II (C2-BIOC, Sigma-Aldrich, United States) for 10 min under identical conditions. Digestion was terminated with 10% fetal bovine serum (FBS) (A3160802, Gibco, United States)-containing DMEM/F12 (11320033, Gibco, United States). Cell suspensions were filtered through 70 μm nylon mesh (352,350, Corning, United States), centrifuged at 300 × g for 5 min, and resuspended in complete medium: DMEM/F12 supplemented with 10% FBS, 1% penicillin-streptomycin (15,140,122, Gibco, United States), and 25 μg/mL ascorbic acid (A4544, Sigma-Aldrich, United States). Primary NPCs were maintained at 37 °C in 5% CO_2_ with medium changes every 48 h until 80% confluency.

### Culture of rat NPCs under different osmotic pressure environments

NPCs were resuspended in DMEM/F12 medium supplemented with 10% FBS and 1% penicillin-streptomycin, and then incubated in a humidified incubator with standard atmospheric conditions (37 °C, 21% O_2_, and 5% CO_2_). The standard culture medium provides an osmolarity of approximately 330 mOsm/kg, which is commonly used as the isotonic control baseline in vitro NPC studies. To establish different osmotic pressure conditions, sodium chloride (NaCl) (S805280, Macklin, China; 9.7 g/L or 16.2 g/L) was dissolved in the culture medium to attain osmolality levels of 330 mOsm/kg (physiological level) and 550 mOsm/kg (hyperosmolarity level). The final osmolality was quantitatively verified through triplicate measurements using a freezing-point osmometer (FM-8P, Shanghai Medical College Instrument Co., Ltd., China).

### Detection of apoptosis in NPCs by flow cytometry

NPCs were seeded in a 6-well plate and exposed to media with osmolarities of 330 or 550 mOsm/kg for 48 h (37 °C and 5% CO_2_). After treatment, the cells were enzymatically dissociated using 0.25% trypsin, washed twice with PBS, and collected by centrifugation at 300 × g for 5 min at 4 °C. Apoptosis analysis was conducted using the Annexin V-FITC/PE Apoptosis Detection Kit (KGA1102, KeyGEN BioTECH, China), following the manufacturer’s protocol. In brief, cells were resuspended at a concentration of 1 × 10^5 cells/mL in 100 μL binding buffer and dual-stained with 5 μL of Annexin V-FITC and 10 μL of PE for 15 min at 25 °C in light-protected conditions. Quantitative flow cytometric analysis was conducted using a FACSCalibur system (BD Biosciences, CA). Fluorescence signals were detected through 530/30 nm (FITC) and 585/42 nm (PE) bandpass filters, with data acquisition performed using CellQuest Pro software.

### Detection of caspase-3 activity by flow cytometry

NPCs were seeded in a 6-well plate at a density of 1 × 10^^5^ cells per well and maintained in a humidified incubator (37 °C, 5% CO_2_). Upon reaching 80%–90% confluence after 48 h of culture, the cell monolayers were washed twice with buffer. Caspase-3 proteolytic activity was measured using the Caspase-3 Activity Assay Kit (C1168S, Beyotime Biotechnology, China), following the manufacturer’s protocol strictly.

### TUNEL staining

Apoptosis in rat NPCs was assessed using a commercial TUNEL kit (C1086, Beyotime Biotechnology, China). A 100 μL proteinase K working solution was added to the isolated NPCs and incubated at 37 °C for 20 min. The NPCs was rinsed in 1 × PBS for 5 min, repeated three times, and then incubated with 50 μL incubation buffer at 37 °C for 1 h. The DAPI working solution was applied and incubated at 37 °C for 10 min before being coverslipped with buffered glycerin. The sample was imaged under a fluorescence microscope (Leica Microsystems, Germany). The number of TUNEL-positive cells (green) and total nuclei (blue) was counted in three randomly selected fields. The ratio of TUNEL-positive cells to the total number of nuclei was calculated using the cell counting method.

### Western blot assay

Protein expression levels were quantified using Western blot analysis, with β-actin serving as the endogenous loading control. Cellular proteins were extracted from NPCs using ice-cold radioimmunoprecipitation assay (RIPA) lysis buffer (P0013B, Beyotime Biotechnology, China). Protein concentrations were then measured using a bicinchoninic acid (BCA) assay kit (23,225, Thermo Fisher Scientific, United States), following the manufacturer’s protocol. Equal amounts of protein lysates were resolved through 10% sodium dodecyl sulfate-polyacrylamide gel electrophoresis (SDS-PAGE; P0012A, Beyotime Biotechnology, China) and subsequently transferred onto polyvinylidene difluoride (PVDF) membranes (IPVH00010, Merck Millipore, United States) using a semi-dry transfer system. The Primary antibodies used include anti-Bax (1:1,000; ab32503, Abcam, United Kingdom); anti-Bcl-2 (1:1,000; ab194583, Abcam, United Kingdom); anti-type II collagen (1:5,000; ab34712, Abcam, United Kingdom); anti-MMP3 (1:1,000; ab52915, Abcam, United Kingdom); anti-AQP3 (1:1,000; ab125219, Abcam, United Kingdom); anti-mTOR (1:10,000; ab134903, Abcam, United Kingdom); anti-p-mTOR (1:1,000; ab109268, Abcam, United Kingdom); anti-PI3K (1:1,000; 4,249, Cell Signaling Technology, United States); anti-p-PI3K (1:1,000; 17,366, Cell Signaling Technology, United States); anti-Akt (1:2000; 2,920, Cell Signaling Technology, United States); anti-p-Akt (1:2000; 4,060, Cell Signaling Technology, United States); anti-β-actin (1:5,000; ab8226, Abcam, United Kingdom). After incubating the primary antibodies overnight at 4 °C, the membranes were washed three times with Tris-buffered saline containing 0.1% Tween-20 (TBST; ST671, Beyotime Biotechnology, China), with gentle agitation. Subsequently, the membranes were probed with species-matched horseradish peroxidase (HRP)-conjugated secondary antibodies (Goat anti-mouse IgG (1:2000; ab6789, Abcam, United Kingdom) and Goat anti-rabbit IgG (1:2000; ab6721, Abcam, United Kingdom) at room temperature for 2 h, with continuous shaking. After three additional TBST washes, immunoreactive bands were visualized using an enhanced chemiluminescence (ECL) kit (A38556, Thermo Fisher Scientific, United States). Protein band intensities were quantified through densitometric analysis with ImageJ software (National Institutes of Health, United States) and normalized to β-actin expression levels.

### Detection of ROS levels in NPCs using flow cytometry

Intracellular reactive oxygen species (ROS) levels were quantified using the 2′,7′-dichlorodihydrofluorescein diacetate (DCFDA) Cellular ROS Detection Assay Kit (ab113851, Abcam, United Kingdom), with protocol optimization. Briefly, NPCs were incubated with 10 μM DCFDA in serum-free RPMI-1640 medium at 37 °C under a 5% CO_2_ workstation for precise environmental control. Following 30 min dark incubation, cells were washed twice with thermostated PBS and resuspended in phenol red-free DMEM/F12 to minimize autofluorescence. Data were acquired using BD FACSDiva software with a threshold set at FSC-H 200. The 2′,7′-dichlorofluorescein (DCF) mean fluorescence intensity (MFI) was quantified after spectral overlap compensation using single-stained controls. ROS levels were expressed as fold change relative to vehicle-treated controls.

### Detection of ROS levels in NPCs by immunofluorescence

Mitochondrial ROS were quantified using a dual-fluorescence colocalization assay. NPCs cultured under osmotic pressure of 330 and 550 mOsm/kg were co-stained with the MitoSOX Red mitochondrial superoxide indicator (M36008, Thermo Fisher Scientific, MA) and MitoTracker Green FM (C1048, Beyotime Biotechnology, China), following the manufacturers’ protocols with optimization. Briefly, cells were incubated with the dye mixture in Hank’s Balanced Salt Solution at 37 °C under 5% CO_2_ for 30 min, followed by three washes with pre-warmed HBSS. Live-cell imaging was performed using an inverted fluorescence microscope (Leica DMi8 Microsystems, Germany). Image acquisition was conducted using LAS X software with identical exposure settings. Colocalization analysis was performed using ImageJ.

### Assessment of GPx and MDA levels

Malondialdehyde (MDA) concentrations and glutathione peroxidase (GPx) activity were quantified using standardized colorimetric and enzymatic assays, respectively. The MDA levels were determined via the thiobarbituric acid reactive substances (TBARS) method, employing a commercial kit (RK09070, ABclonal, China). GPx activity was measured through NADPH oxidation kinetics with a kit (EEA010, Thermo Fisher Scientific, MA). To obtain the supernatant fractions, cell lysates were centrifuged at 12,000 × g for 15 min at 4 °C to obtain supernatant fractions. Absorbance readings were taken with a Synergy H1 microplate reader. The lower detection limits were confirmed to be 0.1 μM for MDA and 5 U/mL for GPx, following serial dilutions of reference standards.

### MMP assessment

Mitochondrial depolarization was evaluated using the JC-1 assay kit (C2006, Beyotime Biotechnology, China), following established protocols. The treated NPCs were incubated with the JC-1 working solution at 37 °C in the dark for 30 min. After being washed three times with PBS, the cells were resuspended in fresh complete medium and immediately analyzed using an inverted fluorescence microscope (DMi8, Leica Microsystems, Germany). Mitochondrial integrity was determined by quantifying the ratio of red to green fluorescence intensity.

Quantitative MMP measurement was performed using JC-1 staining in conjunction with flow cytometry. NPCs induced into apoptosis were harvested, washed with ice-cold PBS, and then resuspended in 1× assay buffer containing JC-1. Following a 20 min incubation at 37 °C in the dark, the cells were centrifuged and washed twice with warm PBS. Cellular fluorescence was immediately analyzed using a FACSCalibur flow cytometer (BD Biosciences, United States). Fluorescence compensation was performed using single-stained controls. The distinct separation of JC-1 monomer and J-aggregate populations, along with the consistent response to osmotic stress, validated the assay’s performance.

### RNA sequencing

RNA extraction from NPCs was performed using TRIzol (15596026, Thermo Fisher Scientific, United States), followed by determination of RNA concentration and purity with a Nanodrop2000 microspectrophotometer. The integrity of RNA samples was examined using the Labchip GX touch microfluidic capillary system. The raw RNA sequencing data underwent quality control (QC) to evaluate their suitability for subsequent analysis. RNA sequencing was conducted on the Illumina HiSeq platform, employing next-generation sequencing (NGS) technology. To assess the functionality of candidate targets in IVDD, Gene Ontology (GO) and Kyoto Encyclopedia of Genes and Genomes (KEGG) pathway enrichment analysis of co-expressed genes was conducted using the clusterProfiler software package in R software. GO and KEGG pathways with P-values less than 0.05 were considered significantly enriched.

### Transfection of expression vectors into NPCs: AAV vector construction and transfection

The pAAV-AQP3 expression plasmid was engineered by subcloning the full-length AQP3 cDNA into the multiple cloning sites of the pAAV-MCS vector (Agilent Technologies, United States) under the control of the CMV immediate-early promoter. The orientation of the insert and the fidelity of the sequence were confirmed through bidirectional Sanger sequencing (GENEWIZ, China), using vector-specific primers (forward: 5′-CGC​AAA​TGG​GCG​GTA​GGC​GTG-3'; reverse: 5′-CTC​AGT​TGG​CGA​GCT​CGG​ATC-3′). NPCs were seeded at a density of 2.0 × 10^^5^ cells/well on poly-L-lysine-coated glass coverslips (0111520, Thermo Fisher Scientific, United States) within 6-well plates (140675, Nunc, Denmark) and cultured overnight in DMEM/F-12 medium supplemented with 10% FBS. Transfection complexes were prepared by combining 2.5 μg of the pAAV-AQP3 plasmid with 7.5 μL of Lipofectamine 2000 (11668019, Thermo Fisher Scientific, United States) in Opti-MEM reduced serum medium (31985062, Gibco, United States), adhering to a 3:1 lipid-to-DNA ratio. After a 20 min incubation at room temperature, the complexes were added to cells in antibiotic-free medium. Following a 6 h incubation at 37 °C with 5% CO_2_, the transfection medium was replaced with complete growth medium, which was maintained for 48 h before subsequent analyses.

### AAV-mediated expression of AQP3

Recombinant adeno-associated virus (rAAV) vectors were generated through triple-plasmid co-transfection in HEK293T cells (CRL-3216, ATCC, United States). The payload plasmid, pAAV-TBG-AQP3-GFP, contained human AQP3 cDNA fused with an enhanced GFP reporter, a hepatocyte-specific thyroxine-binding globulin (TBG) promoter, and AAV2 inverted terminal repeats (ITRs). The packaging system consisted of pAAV-RC2/8, which provided AAV2 replication (Rep) and AAV8 capsid (Cap) proteins, and pHelper, which supplied adenoviral E2A, E4, and VA RNA genes.

At 72 h post-transfection, the cells were lysed using 0.5% sodium deoxycholate (D6750, Sigma-Aldrich, United States) and then subjected to cesium chloride gradient ultracentrifugation (Optima XE-90, Beckman Coulter, United States) at 210,000 × g for 48 h at 4 °C. Viral fractions with refractive indices ranging from 1.365 to 1.371 were collected and dialyzed against PBS containing 5% sorbitol (S3889, Sigma-Aldrich, United States). Genome titers (1.2 × 10^13 vg/mL) were determined by absolute quantification using ITR-targeted qPCR (Forward: 5′-AGA​AGG​AGT​TGA​TGA​ACC​GTT​GCG-3′; Reverse: 5′-AAC​CAC​AGC​CGA​ACA​TCA​CAA​GG-3′) with linearized plasmid standards.

### Animal experiment

Forty 12-weeks-old female Sprague-Dawley rats were randomly assigned to four experimental groups using stratified randomization based on body weight: Control, Annulus fibrosus puncture (AFP), AFP + AQP3 inhibitor DFP00173 (HY-126073, MedChemExpress, China), and AFP + AAV-AQP3.

The Animals were anesthetized with an intraperitoneal injection of sodium pentobarbital (40 mg/kg; P3761, Sigma-Aldrich, United States) and positioned prone on a warming pad. Under fluoroscopic guidance (C-arm Ziehm Vision RFD 3D, Germany), the caudal intervertebral disc (Co6/7 level) was accessed through a posterolateral approach. A 26-gauge spinal needle (405210, B. Braun, Germany) was inserted perpendicular to the annulus fibrosus at a depth of 5 mm, followed by 360° rotation with a 30-s dwell time to induce a controlled annular injury.

DFP00173 (20 mg/kg) or an AAV-AQP3 solution (1 × 10^^9^ TU/mL AAV vectors) was injected intradiscally using a microsyringe. Postoperative analgesia was maintained with meloxicam (1 mg/kg SC; M3937, Sigma-Aldrich, United States) for 72 h. Booster injections were administered weekly under isoflurane anesthesia. Body weights were recorded biweekly using calibrated scales (XB220A, Precisa, Switzerland).

### Micro-CT and MRI

After a 5-weeks recovery period post-surgery, the animals were subjected to *in vivo* imaging while under isoflurane anesthesia (3% for induction and 1.5% for maintenance). High-resolution micro-computed tomography (micro-CT) scans were obtained using a SkyScan 1,276 system (Bruker, Germany).

T2-weighted imaging was performed using a 3T clinical scanner (MAGNETOM Prisma; Siemens Healthineers, Germany) equipped with a 16-channel phased-array spine coil. The Sequence parameters were optimized for intervertebral disc assessment (repletion time 2000 ms; echo time 76 ms; field of view 260 × 320 mm; slice thickness 0.8 mm).

### Histopathologic staining

IVD specimens were fixed in 4% paraformaldehyde (PFA; P6148, Sigma-Aldrich, United States) for 48 h at 4 °C, followed by decalcification in a 10% ethylenediaminetetraacetic acid (EDTA; E4884, Sigma-Aldrich, United States) solution for 28 days. The Tissues were dehydrated through a graded ethanol series (70%–100%), cleared in xylene (534056, Sigma-Aldrich, United States), and embedded in paraffin (76242, Leica, Germany) using a HistoStar embedding station (Thermo Fisher Scientific, MA).

Serial 5 μm sagittal sections were cut using an RM2255 microtome (Leica) and mounted on poly-L-lysine-coated slides (S8902, Solarbio, China). The sections were stained with Mayer’s hematoxylin (HXG732, Baso, China) for 8 min and eosin Y (HT110232, Sigma-Aldrich, United States) for 1 min. The sections were then stained with 0.1% Safranin O (S8884, Sigma-Aldrich, United States) for 5 min, followed by 0.02% Fast Green FCF (FCF-1, Sigma-Aldrich, United States) for 3 min. Blinded histological analysis was performed by two independent pathologists using a histological grading scale system (Masuda et al., 2005).

### Statistical analysis

All graphical representations were generated using GraphPad Prism v8.0.2 (GraphPad Software, United States). For continuous data that passed the Shapiro-Wilk normality test, we employed Student’s t-test (for two-group comparisons) or one-way/two-way ANOVA (for multiple groups), followed by Tukey’s *post hoc* test for detailed comparisons. For ordinal data that were not normally distributed, we explicitly stated that the non-parametric Kruskal-Wallis test was used, followed by Dunn’s *post hoc* test for multiple comparisons. The Type I error rate was controlled at α = 0.05 with two-tailed testing. Multiplicity-adjusted p-values are denoted as *P < 0.05 (95% CI excludes null), **P < 0.01, ***P < 0.001. All experiments included at least three independent biological replicates with technical triplicates. Randomization and blinding protocols were implemented during the data collection and analysis phases.

## Results

### Hyperosmolarity inhibited proliferation and induced apoptosis in NPCs

**FIGURE 1:**
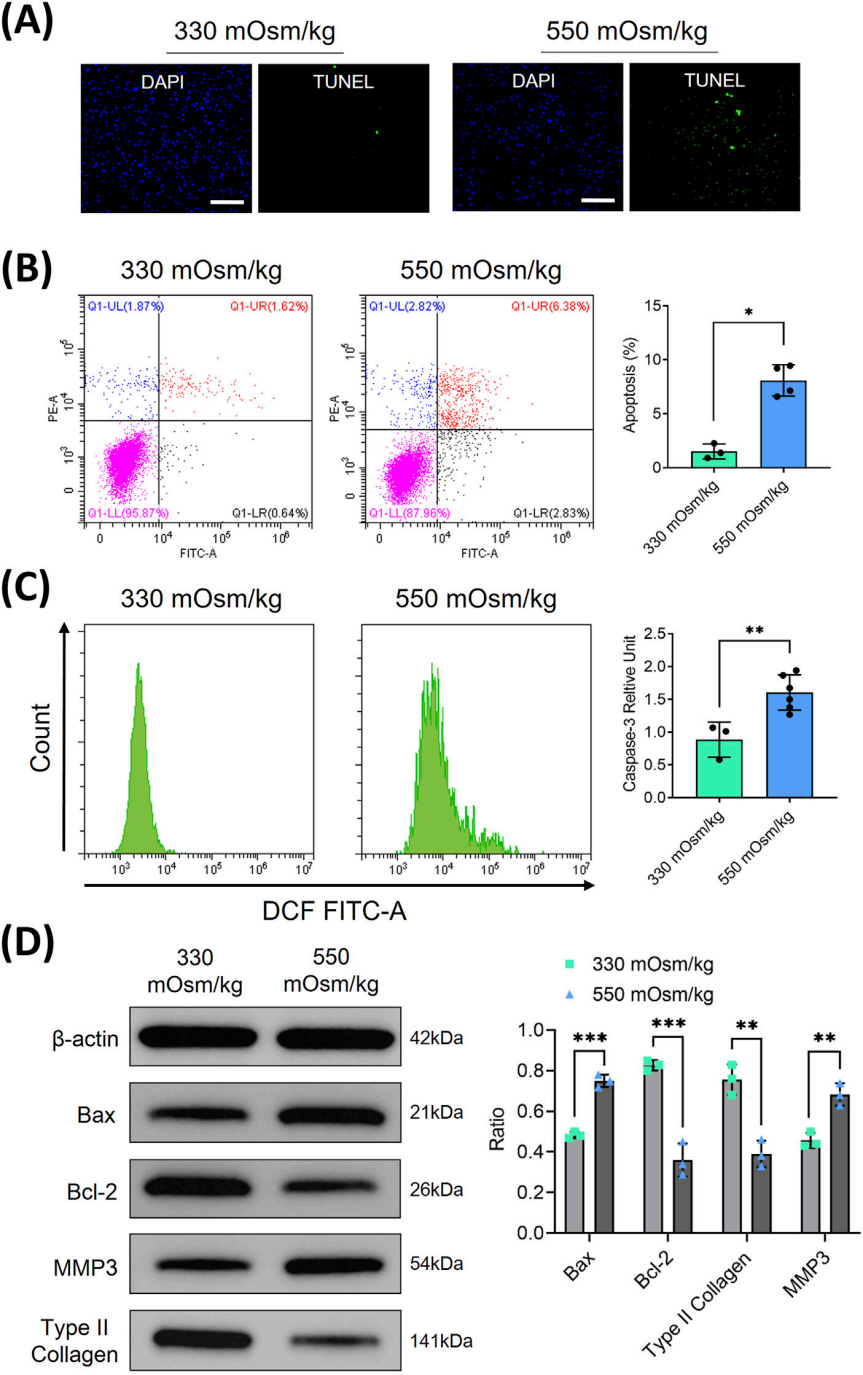
Hyperosmolarity inhibited proliferation and induced apoptosis in rat NPC. **(A)** NPC apoptosis was assessed using TUNEL staining. Scar bar: 100 μm. **(B)** Flow cytometry was employed to quantitatively analyze the apoptosis rates of NPCs under different osmotic pressures (330 and 550 mOsm/kg). **(C)** Flow cytometry was also used to detect the activation of cleaved caspase-3 in apoptotic NPCs through intracellular staining with specific antibodies. **(D)** Western blot analysis was conducted to evaluate apoptosis-related proteins (Bax, Bcl-2) and extracellular matrix components (type II collagen, MMP3) at osmotic pressures of 330 mOsm/kg and 550 mOsm/kg. Representative immunoblots and densitometric quantification, normalized to β-actin (42 kDa), are presented to illustrate protein expression profiles. Molecular weight markers indicated in kDa. Data are expressed as mean ± SD. Significant differences between groups are indicated as *p < 0.05, **p < 0.01, ***p < 0.001.

The pathogenesis of IVDD has been associated with altered osmotic homeostasis. To investigate the hyperosmolar effects on NPCs, primary rat NPC cultures were established and subjected to graded osmotic stress (330 and 550 mOsm/kg) for 48 h. Quantitative apoptosis assessment through immunofluorescence and flow cytometry revealed substantial cell death potentiation under hyperosmolarity conditions (550 mOsm/kg), with apoptosis rates increased from 1.62% of physiological osmolality controls (330 mOsm/kg) to 6.38% under hyperosmolarity conditions (550 mOsm/kg) (Figures 1A,B). This apoptotic activation was corroborated by significant elevation of cleaved caspase-3 expression, as quantified through flow cytometric analysis (Figure 1C). Mechanistic investigation via Western blotting demonstrated a marked upregulation of pro-apoptotic Bax protein coupled with a concomitant downregulation of anti-apoptotic Bcl-2 in the 550 mOsm/kg group (Figure 1D). Furthermore, ECM degradation was evidenced by significant reductions in type II collagen and an increase in MMP3 expression levels. These findings collectively establish that sustained hypertonic stress induces apoptotic cascades in NPCs while disrupting matrix homeostasis.

### Hyperosmolality induces mitochondrial oxidative damage and ROS accumulation in NPCs

**FIGURE 2:**
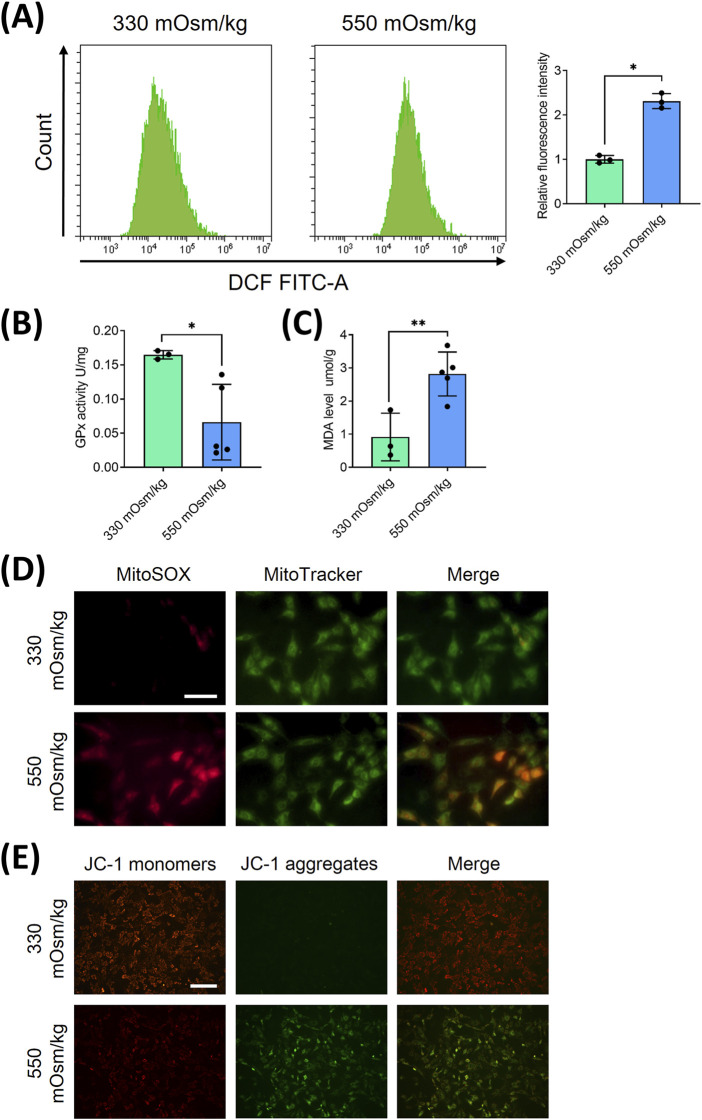
Hyperosmolality induces mitochondrial oxidative damage and ROS accumulation in NPCs. **(A)** Flow cytometric quantification of intracellular ROS levels under osmotic pressures of 330 mOsm/kg and 550 mOsm/kg. **(B)** The activity of GPx, measured by colorimetric assay, indicates enzymatic antioxidant capacity attenuation under different osmotic pressures (330 mOsm/kg and 550 mOsm/kg). **(C)** Quantification of MDA content through the thiobarbituric acid reaction reflects lipid peroxidation levels under different osmotic pressures (330 mOsm/kg and 550 mOsm/kg). **(D)** Confocal imaging of mitochondrial superoxide (MitoSOX, Red) and mitochondrial morphology (MitoTracker, Green) co-localization. Scar bar: 200 μm. **(E)** MMP assessment using JC-1 fluorescence shift (red/green ratio). Scar bar: 100 μm. Data represent mean ± SD from three biological replicates. Significant differences between different groups are indicated as *p < 0.05, **p < 0.01.

To elucidate the mechanisms underlying hyperosmolarity-induced cellular damage, we investigated the role of reactive oxygen species (ROS) in NPCs, given their established involvement in apoptotic pathways (Cheng et al., 2025). Flow cytometric analysis revealed distinct alterations in oxidative stress markers across different osmotic conditions. Specifically, NPCs exposed to 550 mOsm/kg exhibited a marked elevation in ROS levels (Figure 2A), accompanied by a significant reduction in GPx activity (Figure 2B) and increased MDA content (Figure 2C), compared to the 330 mOsm/kg control group.

Considering the mitochondrial origin of ROS generation, we employed dual immunofluorescence staining using Mito-SOX (red) and Mito-Tracker (green) to specifically assess mitochondrial ROS production. Intracellular and mitochondrial ROS staining showed that both Mito-Tracker (green) and Mito-SOX (red) fluorescent levels were increased in NPCs with 550 mOsm/kg (Figure 2D), indicating heightened mitochondrial oxidative stress. To further evaluate mitochondrial integrity, we examined the mitochondrial membrane potential (MMP), a critical parameter of mitochondrial function, using JC-1 staining. MMP was detected by JC-1 staining, which shows red fluorescence in normal mitochondria and green fluorescence in cases of mitochondrial dysfunction. Hyperosmolarity increased the green fluorescence and decreased red fluorescence, indicating a decline in MMP (Figure 2E). These collective findings strongly suggest that hyperosmolarity induces mitochondrial dysfunction in NPCs, leading to excessive ROS accumulation and subsequent cellular damage.

### The overexpression of AQP3 alleviated mitochondrial oxidative stress and reduced ROS accumulation in NPCs subjected to high osmotic pressure

**FIGURE 3:**
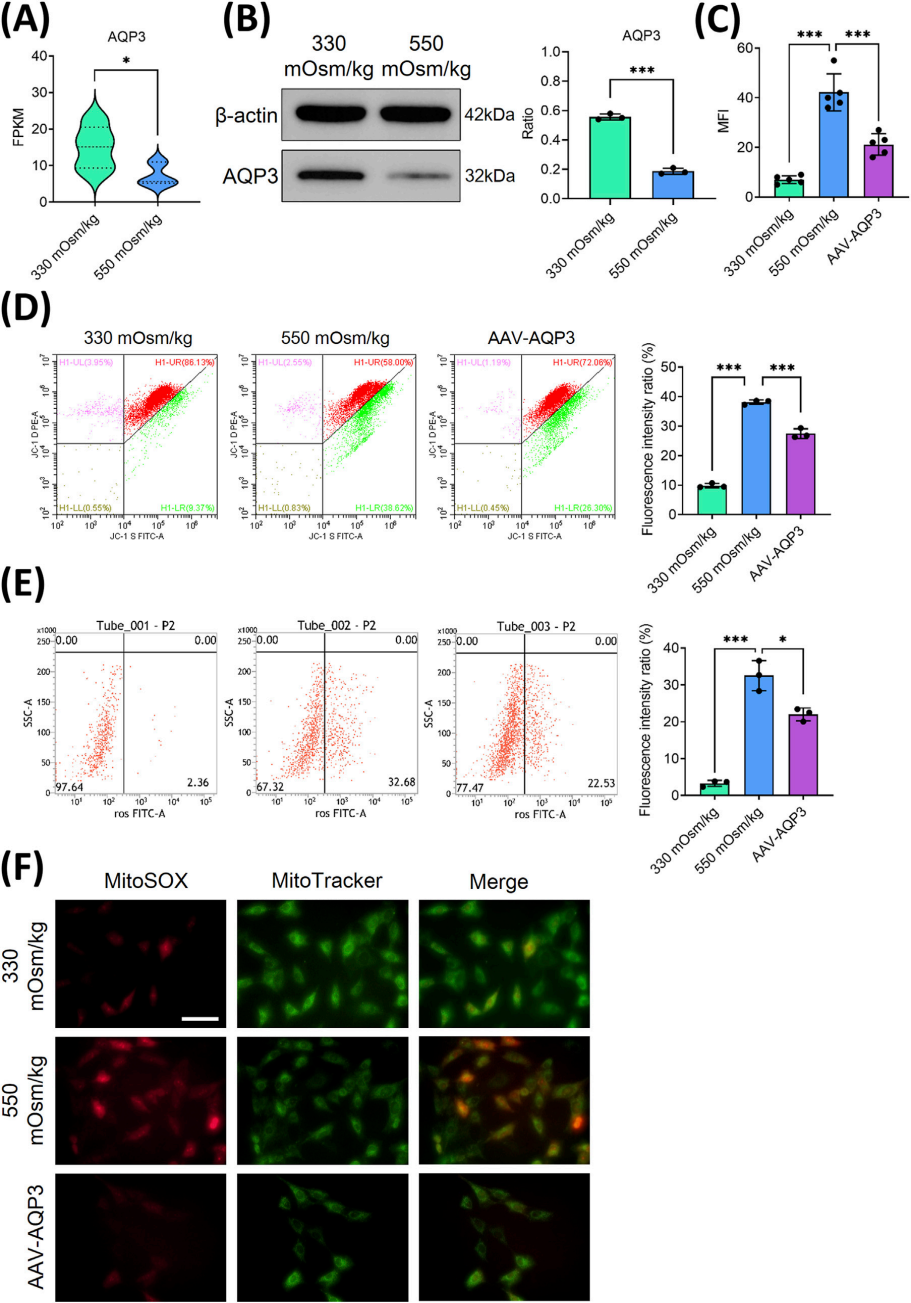
The overexpression of AQP3 alleviated mitochondrial oxidative stress and reduced the accumulation of ROS in NPCs subjected to high osmotic pressure. **(A)** The mRNA expression of AQP3 in NPCs exposed to different osmotic pressures (330 mOsm/kg and 550 mOsm/kg). **(B)** Western blot analysis of AQP3 protein levels under different osmotic pressures (330 mOsm/kg and 550 mOsm/kg). Representative blots and densitometric quantification, normalized to β-actin (42 kDa), are presented. **(C)** Quantitative analysis of apoptotic NPCs. **(D)** MMP preservation, as assessed by flow cytometry (green dots), in NPCs of each group. **(E)** Intracellular ROS levels measured using flow cytometry. **(F)** Confocal microscopy images showing the co-localization of mitochondrial superoxide (MitoSOX, red) and mitochondrial morphology (MitoTracker, green). Scar bar: 200 μm. Data are presented as the mean ± SD from three biological replicates. Significant differences between different groups are indicated as *p < 0.05, ***p < 0.001.

AQP3, a member of the aquaporin family responsible for the transport of water and small solutes, is significantly decreased in aging IVD (Xie et al., 2016). To explore its function under hyperosmotic conditions, we examined AQP3 expression in NPCs. RNA sequencing analysis indicated a substantial decrease in AQP3 expression in NPCs subjected to 550 mOsm/kg compared to those exposed to 330 mOsm/kg (Figure 3A). In agreement with these findings, Western blot analysis showed notably reduced AQP3 protein levels under hyperosmotic conditions (Figure 3B), suggesting that osmotic stress inhibits AQP3 expression.

To evaluate the functional impact of AQP3 on NPCs survival, lentiviral AQP3 overexpression (AAV-AQP3) was performed. Flow cytometry analysis revealed that AAV-AQP3-transfected NPCs exhibited a lower apoptotic rate under 550 mOsm/kg compared to controls (Figure 3C). Concurrently, JC-1 staining indicated an elevated MMP (Figure 3D), while ROS accumulation was significantly attenuated in the AAV-AQP3 group (Figure 3E). Immunofluorescence further confirmed a reduction in Mito-SOX fluorescence intensity in AQP3-overexpressing NPCs (Figure 3F). Collectively, these findings demonstrate that AQP3 overexpression alleviates mitochondrial dysfunction, suppresses ROS production, and enhances NPCs viability under hyperosmotic conditions.

### Downregulation of AQP3 exacerbates NPCs apoptosis through inhibition of the PI3K/Akt/mTOR pathway under hyperosmolar conditions

**FIGURE 4:**
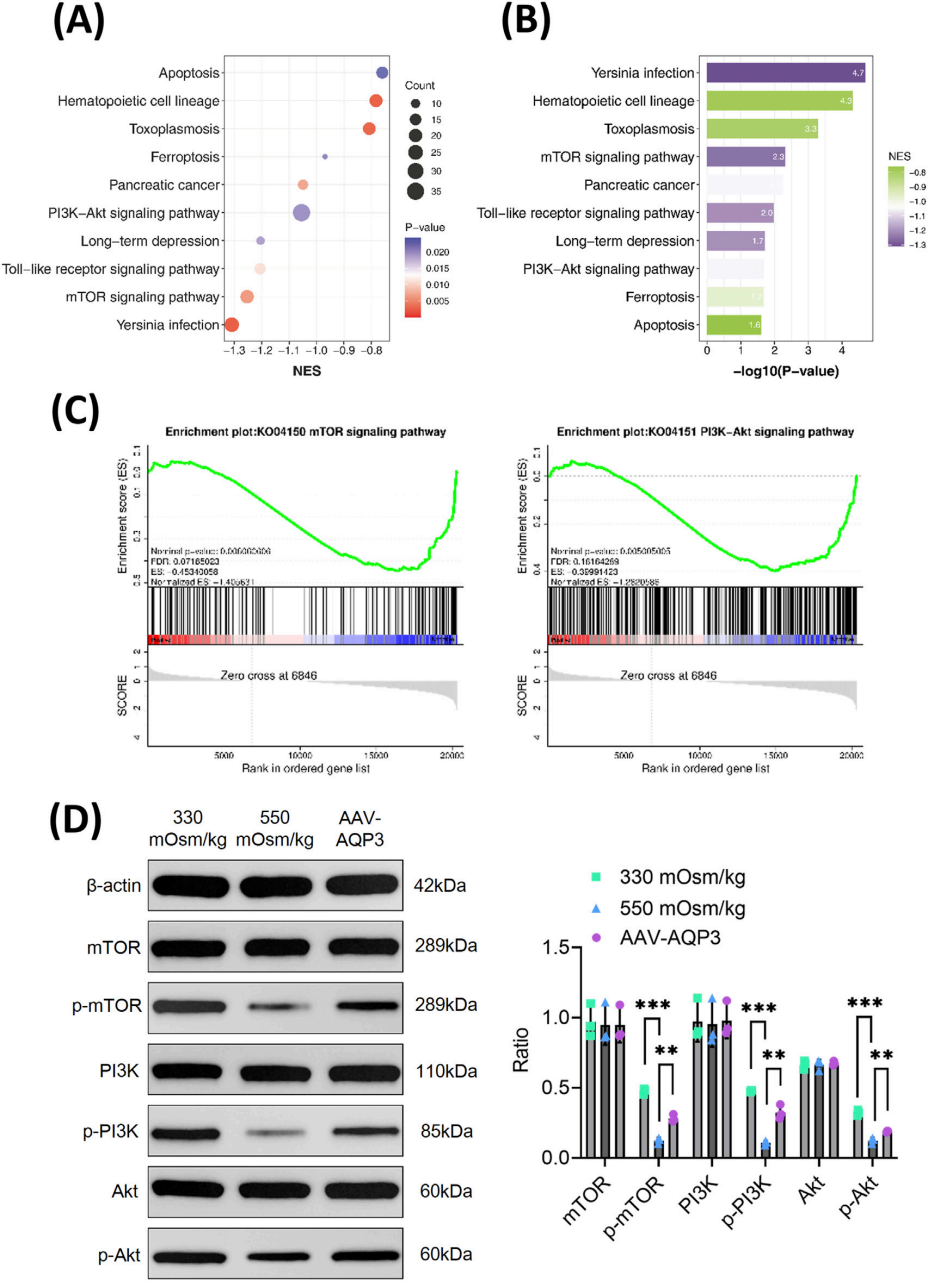
The downregulation of AQP3 intensifies the apoptosis of NPCs by inhibiting the PI3K/Akt/mTOR pathway under hyperosmotic conditions. **(A)** GSEA of selected KEGG pathways enriched in the 550 mOsm/kg group compared to the 330 mOsm/kg group for NPCs. Spot diameter indicates gene count, while the color corresponds to the log10-transformed p value. **(B)** KEGG pathways enriched with genes downregulated in the 550 mOsm/kg group relative to the 330 mOsm/kg group for NPCs. **(C)** GSEA showing the coordinated suppression of PI3K/Akt and mTOR signaling components. The y-axis indicates the enrichment score, and the x-axis represents the genes within the gene sets. **(D)** Western blot analysis of p-mTOR, p-PI3K, and p-Akt level in rat NPCs from each group. Data are presented as mean ± SD from three biological replicates. Significant differences between groups are indicated as **p < 0.01, ***p < 0.001.

To elucidate the mechanism by which AQP3 downregulation contributes to NPCs apoptosis, RNA sequencing and pathway enrichment analyses were conducted. To ascertain the role and function of NPCs from the 550 mOsm/kg group, a differential expressionanalysis was performed comparing the 330 mOsm/kg and 550 mOsm/kg groups. The differentially expressed genes (DEGs) were then subjected to gene set enrichment analysis (GSEA). GSEA indicated that pathways such as PI3K/Akt and mTOR signaling pathways were significantly downregulated in NPCs exposed to 550 mOsm/kg (Figures 4A–C). Western blot analysis confirmed decreased phosphorylation levels of PI3K, Akt, and mTOR under hyperosmotic conditions. Interestingly, AQP3 overexpression restored their expression (Figure 4D), suggesting that AQP3 modulates these pathways. These findings indicate that hyperosmolarity-induced AQP3 depletion promotes NPC apoptosis by disrupting the PI3K/Akt/mTOR pathway.

### Enhanced expression of AQP3 mitigated the progression of IVDD in the rat model

**FIGURE 5:**
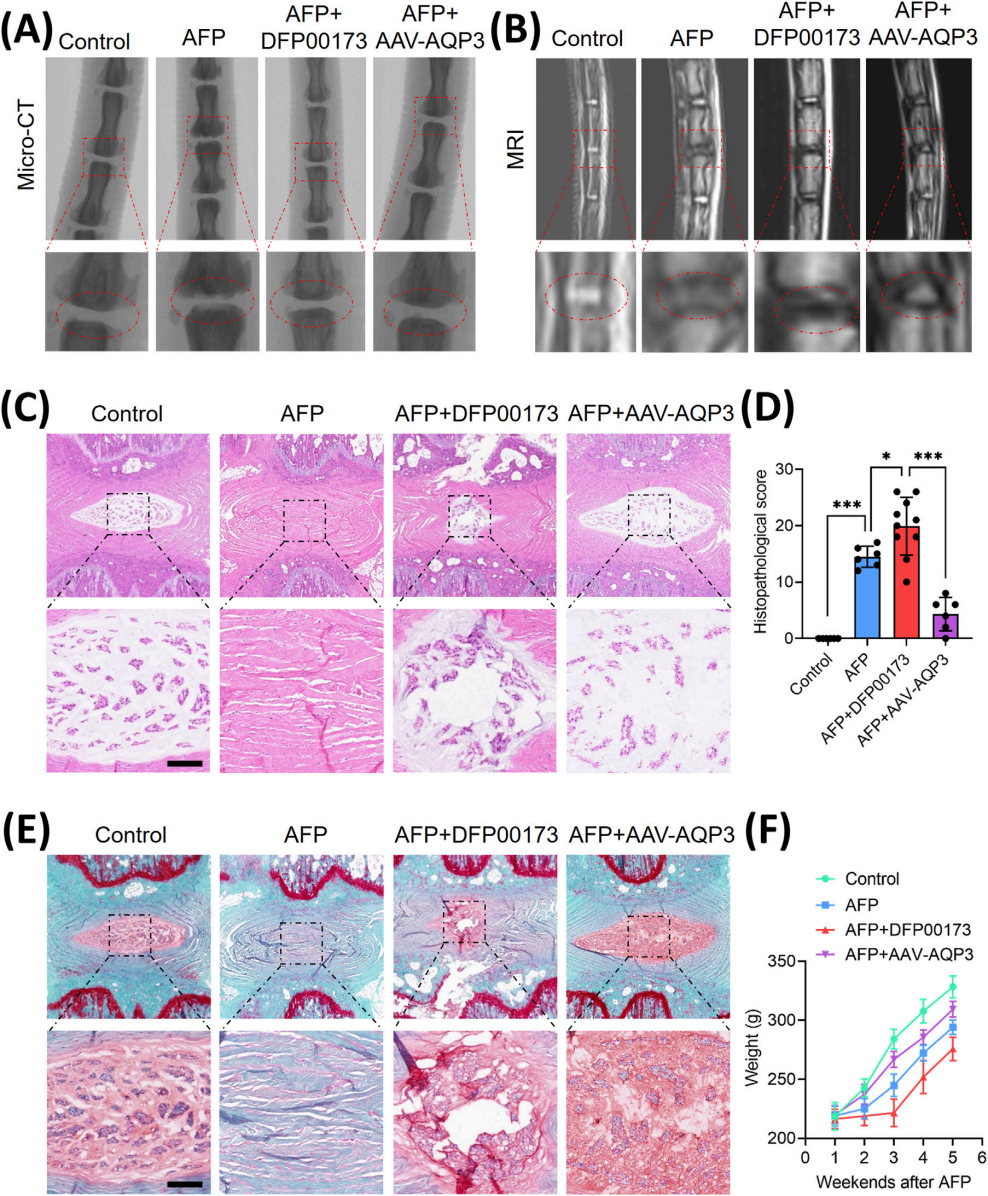
The overexpression of AQP3 alleviated the progression of IVDD in rat model. **(A)** Schematic representation of the experimental design: Control (n = 6), AFP (n = 6), AFP + DFP00173 (n = 10), and AFP + AAV-AQP3 (n = 6) groups. Interventions were administered via intradiscal injection weekly for 5 weeks post-puncture. Micro-CT sagittal reconstructions demonstrate the preservation of intervertebral space height. **(B)** T2-weighted MRI axial sections. The operated levels (Co6/7) are demarcated by white triangles. **(C–E)** Histopathological evaluation of NP tissue integrity. Representative H&E staining and Safranin-O/fast green staining with semi-quantitative histomorphometric analysis. Scale bar: 500 μm. **(F)** Average growth weight curves of rats in each group. Data represent mean ± SD. Significant differences between different groups are indicated as *p < 0.05, ***p < 0.001.

To validate the therapeutic potential of AQP3 *in vivo*, an IVDD model was established via annulus fibrosus puncture (AFP). Rats received weekly injections of the AQP3 inhibitor (DFP00173) or AAV-AQP3. After 5 weeks, micro-CT imaging revealed narrowed intervertebral disc spaces in IVDD rats, which were partially restored by AQP3 overexpression (Figure 5A). MRI analysis indicated uneven disc intensity and diminished T2-weighted signals in IVDD rats, whereas treatment with AAV-AQP3 elevated T2 signals (Figure 5B), suggesting improved disc hydration.

Histopathological assessment demonstrated severe structural degeneration in AFP-induced IVDD rats, including collapsed disc height, NPCs loss, and fibrosis. In contrast, administration of AAV-AQP3 ameliorated tissue architecture, reduced fibrosis, and lowered histological scores (Figures 5C–E). Furthermore, rats overexpressing AQP3 exhibited improved weight gain compared to the IVDD group (Figure 5F). These results confirm that AQP3 overexpression mitigates IVDD progression *in vivo*.

## Discussion

LBP secondary to IVDD is a leading cause of global disability. This degenerative process is driven by multiple risk factors, including advancing age, genetic predisposition, chronic inflammation, immune dysfunction, hyperosmolarity, and nutritional deficiency. These stimuli induce apoptosis of NPCs and disruption of AF cells, leading to intervertebral space narrowing, NP herniation, and nerve root or spinal cord compression—hallmarks of chronic pain (Knezevic et al., 2021; Risbud and Shapiro, 2014). IVDD involves deregulated ECM synthesis, increased catabolism accompanied by cellular senescence, and apoptosis-mediated loss of nucleus pulposus cells (Silagi et al., 2018; Choi et al., 2018; Tessier et al., 2020). Notably, microenvironmental hyperosmolarity has been identified as a critical modulator of NPCs. Studies show that exposing NPCs to 550 mOsm/kg osmolarity induces apoptosis, evidenced by nuclear fragmentation, chromatin condensation, and organelle breakdown. In contrast, physiological osmolarity of approximately 450 mOsm/kg-comparable to native NP tissue-exerts minimal apoptotic effects on cell viability (Li et al., 2017).

Emerging evidence suggests that osmotic stress mediates the dysregulation of AQP3 in NPCs, with previous studies empirically demonstrating a 40% reduction in AQP3 expression at 550 mOsm/kg compared to physiological 330 mOsm/kg conditions (Zhang et al., 2022). Although osmotic sensitivity has been documented, the connection between AQP3 downregulation and NPC apoptosis remains unclear. We systematically evaluated apoptotic dynamics and AQP3 expression across osmotic gradients and linked these changes to ROS-mediated mitochondrial dysfunction via inhibition of the PI3K/Akt/mTOR pathway. Three principal findings emerge from this study: first, hyperosmolarity (550 mOsm/kg) induces significant AQP3 suppression, correlating with elevated intracellular ROS levels and increased NPC apoptosis. Second, pharmacological overexpression of AQP3 restores redox homeostasis-enhancing mitochondrial membrane potential stability and reducing caspase-3 activation. Third, pathway analysis reveals that hyperosmolarity disrupts the PI3K/Akt/mTOR pathway, and phosphorylated Akt levels decrease proportionally with osmotic intensity.

The clinical data emphasize the pathophysiological significance of these findings, indicating a 60% reduction in AQP3 expression in IVDD patient specimens compared to healthy controls (Li et al., 2014). AQP3, an aquaglyceroporin, facilitates the transport of both water and glycerol and also contributes to cellular antioxidant defenses (Chen et al., 2011). The intracellular glycerol pool maintained by AQP3 may influence phosphoinositide metabolism, potentially affecting PIP3 generation - a key second messenger for PI3K/Akt pathway activation. Moreover, emerging evidence indicates that AQP3 can transport hydrogen peroxide (H_2_O_2_), which at physiological concentrations acts as a signaling molecule that can oxidize and inhibit PTEN, the primary negative regulator of the PI3K pathway. Therefore, AQP3 downregulation under hyperosmotic stress may disrupt this redox signaling axis, leading to sustained PTEN activity and consequent suppression of PI3K/Akt/mTOR signaling. (Hara-Chikuma et al., 2015; Wang et al., 2021). Additionally, the osmotic stress-induced disruption of AQP3-mediated water transport may affect membrane tension and organization, potentially influencing the spatial organization and activation of membrane-associated signaling complexes including those involving PI3K. These proposed mechanisms provide testable hypotheses for future investigations into the precise molecular links between AQP3 function and PI3K/Akt/mTOR pathway regulation. Our results pinpoint AQP3 as a crucial mediator of osmotic adaptation, redox balance, and apoptotic signaling in the pathogenesis of IVDD.

Our experimental data reveal a distinct osmotic threshold response in NPCs, where an osmotic pressure of 550 mOsm/kg induces increased apoptosis rates and diminishes proliferative capacity compared to physiological 330 mOsm/kg conditions. This corroborates previous findings by Li’s group on the osmotic sensitivity of disc cells (Zhang et al., 2022). Hyperosmolarity triggers mitochondrial oxidative damage, driving a cycle of ROS accumulation and cellular dysfunction. Inflammatory mediators and inflammatory cells recruited by neovascularization promote excessive ROS generation, thereby inducing oxidative stress (Suzuki et al., 2015). This oxidative stress damages crucial intracellular macromolecules, including lipids, DNA (both nuclear and mitochondrial), and proteins, ultimately accelerating cellular dysfunction and tissue degeneration (Zhang et al., 2025). Vascular endothelial growth factor (VEGF)-induced neoangiogenesis disrupts the normally avascular NP microenvironment, potentially leading to erythrocyte infiltration and the release of iron ions, which catalyze Fenton reactions (Ali et al., 2008). Furthermore, the opening of mitochondrial permeability transition pore (mPTP) opening cytochrome c release and activates the caspase-3-dependent apoptotic pathway (Xu et al., 2019).

Magnesium boride-alginate (MB-ALG) hydrogels have been shown to promote the proliferation of senescent cells within rat IVD through by activating the PI3K/Akt/mTOR signaling cascade. Concurrently, molecular hydrogen has demonstrated remarkable antioxidative efficacy by effectively neutralizing ROS, thereby protecting NPCs from oxidative stress-induced damage (Han et al., 2024). Sun et al. demonstrated that hyperosmolarity suppresses the PI3K/Akt/mTOR pathway and that osteogenic protein-1 (OP-1) reverses this effect, reducing NPC apoptosis (Yang et al., 2018). We subsequently investigated how downregulation of AQP3 contributes to NPC apoptosis under high osmotic pressure. RNA sequencing revealed that hyperosmolar conditions significantly inhibit the PI3K/Akt/mTOR signaling axis in rat nucleus pulposus cells. Importantly, overexpression of AQP3 mediated by an adenoviral vector successfully reversed this pathway suppression, leading to a pronounced decrease in NPCs apoptosis. These molecular interventions translated into significant improvements in the IVDD rat model, highlighting the therapeutic promise of AQP3 modulation and PI3K/Akt/mTOR pathway targeting for IVDD management.

Previous investigations by Palacio-Mancheno et al. revealed elevated AQP3 expression in notochordal cell lineages following 14-day hyperosmotic conditioning of murine intervertebral disc cell cultures (Palacio-Mancheno et al., 2018), presenting divergent outcomes from our experimental findings. This discrepancy underscores the critical influence of cellular heterogeneity in the intervertebral disc. While our isolation protocol was optimized for mature NPCs, we acknowledge the potential contribution of notochordal cell remnants in primary rodent cultures as a limitation. Nonetheless, several factors bolster the validity of our conclusions: first, the isolated cells exhibited the characteristic rounded, vacuole-rich morphology of mature NPCs, distinct from the larger, cluster-forming notochordal cells; second, their gene expression profile was consistent with mature NPC markers; and finally, the pronounced and consistent downregulation of AQP3 across all isolation batches and replicates strongly indicates this is a predominant response in our NPC population. Employing lineage-specific markers or single-cell RNA sequencing in future work will be crucial to unequivocally delineate these celltype-specific responses.

## Clinical perspectives

• Hyperosmolarity has emerged as a pathogenic driver of NPCs apoptosis during IVDD; however, the underlying mechanism remains unclear.
• Hyperosmolarity triggers NPCs apoptosis by depleting AQP3 and suppressing the PI3K/Akt/mTOR pathway, resulting in mitochondrial dysfunction and ROS accumulation.
• These findings clarify the role of AQP3 in IVDD and provide a molecular basis for developing AQP3-targeted clinical therapies.

## Conclusion

This study elucidates how AQP3 downregulation mediates NPC apoptosis under hyperosmolar condition. Our experimental show that hyperosmolarity significantly increases apoptosis in rat nucleus pulposus cells and decreases AQP3 expression. Notably, AQP3 overexpression rescues NPCs from hyperosmolarity-induced apoptosis by activating the PI3K/Akt/mTOR pathway. These findings clarify the role of AQP3 in IVDD and provide a molecular basis for developing AQP3-targeted clinical therapies.

## Data Availability

The data presented in the study are publicly available. The data are deposited in the Sequence Read Archive (SRA) repository with accession number PRJNA1347831.

## Glossary

LBP: lower back pain
IVDD: intervertebral disc degeneration
IVD: intervertebral disc
NP: nucleus pulposus
AF: annulus fibrosus
ECM: extracellular matrix
NPCs: nucleus pulposus cells
AQPs: aquaporins
AQP3: aquaporin-3
SPF: specific-pathogen-free
FBS: fetal bovine serum
RIPA: radioimmunoprecipitation assay
BCA: bicinchoninic acid
SDS-PAGE: sodium dodecyl sulfate polyacrylamide gel electrophoresis
PVDF: polyvinylidene difluoride
HRP: horseradish peroxidase
ECL: enhanced chemiluminescence
CCK-8: Cell counting kit-8
ROS: reactive oxygen species
DCFDA: 2′,7′-dichlorodihydrofluorescein diacetate
DCF: 2′,7′-dichlorofluorescein
MFI: mean fluorescence intensity
MDA: malondialdehyde
GPx: glutathione peroxidase
TBARS: thiobarbituric acid reactive substances
QC: quality control
NGS: next-generation sequencing
GO: Gene ontology
KEGG: Kyoto encyclopedia of genes and genomes
rAAV: Recombinant adeno-associated virus
TBG: thyroxine-binding globulin
ITRs: inverted terminal repeats
AFP: annulus fibrosus puncture
micro-CT: micro-computed tomography
MRI: magnetic resonance imaging
H&E: hematoxylin and eosin
SD: standard deviation
MMP: mitochondrial membrane potential
DEGs: differentially expressed genes
GSEA: Gene set enrichment analysis
VEGF: vascular endothelial growth factor
mPTP: mitochondrial permeability transition pore
NAC: N-acetylcysteine
MB-ALG: magnesium boride-alginate
OP-1: osteogenic protein-1
