# Supplementary material for: Downregulation of aquaporin 3 promotes hyperosmolarity-induced apoptosis of nucleus pulposus cells through PI3K/Akt/mTOR pathway suppression
Source: Front Genet. 2025 Nov 5;16:1665899. doi: 10.3389/fgene.2025.1665899 (PMC12626380; doi:10.3389/fgene.2025.1665899)

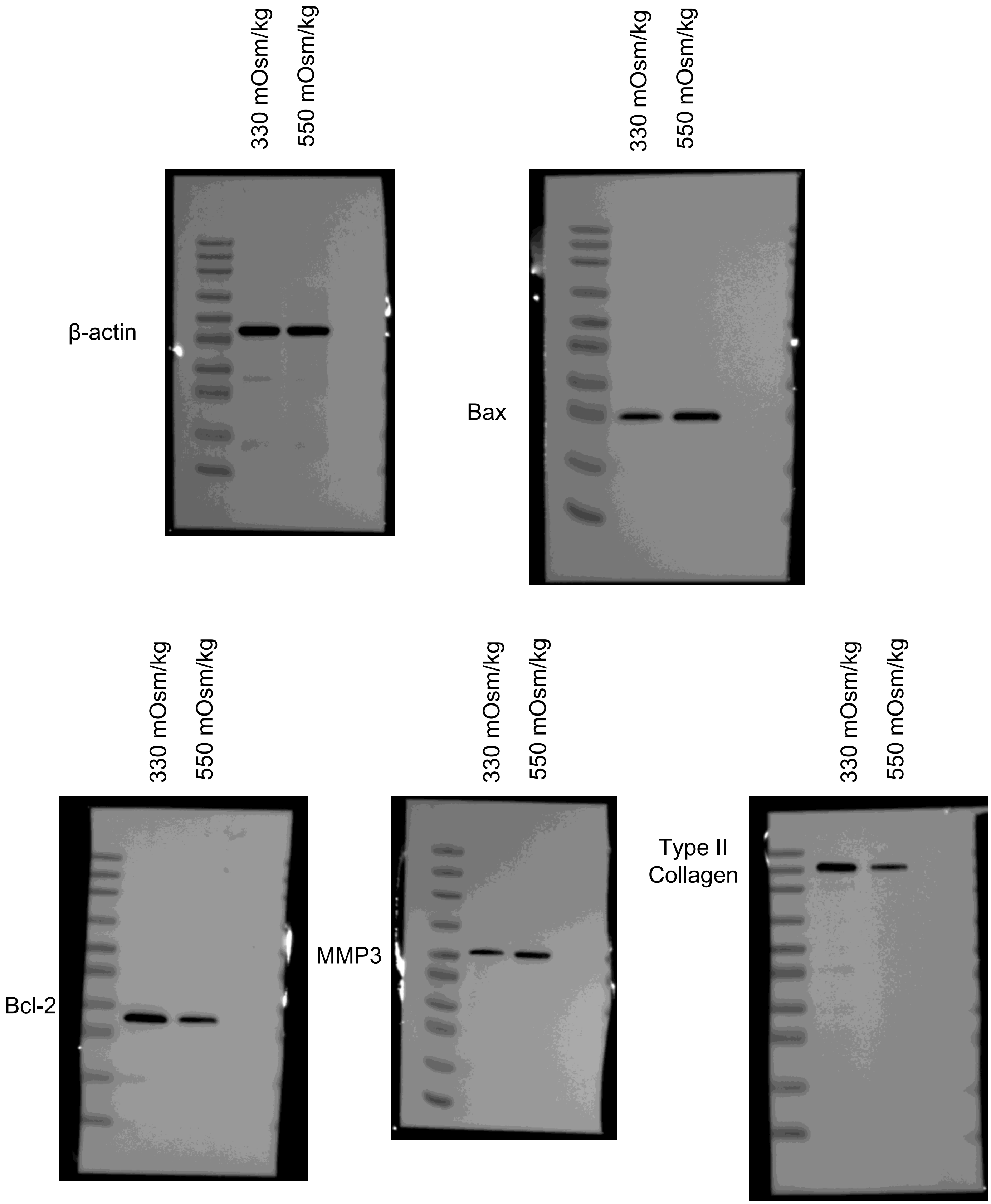
330 mOsm/kg
550 mOsm/kg
β-actin
330 mOsm/kg
550 mOsm/kg
Bax
330 mOsm/kg
550 mOsm/kg
Bcl-2
330 mOsm/kg
550 mOsm/kg
MMP3
330 mOsm/kg
550 mOsm/kg
Type II
Collagen

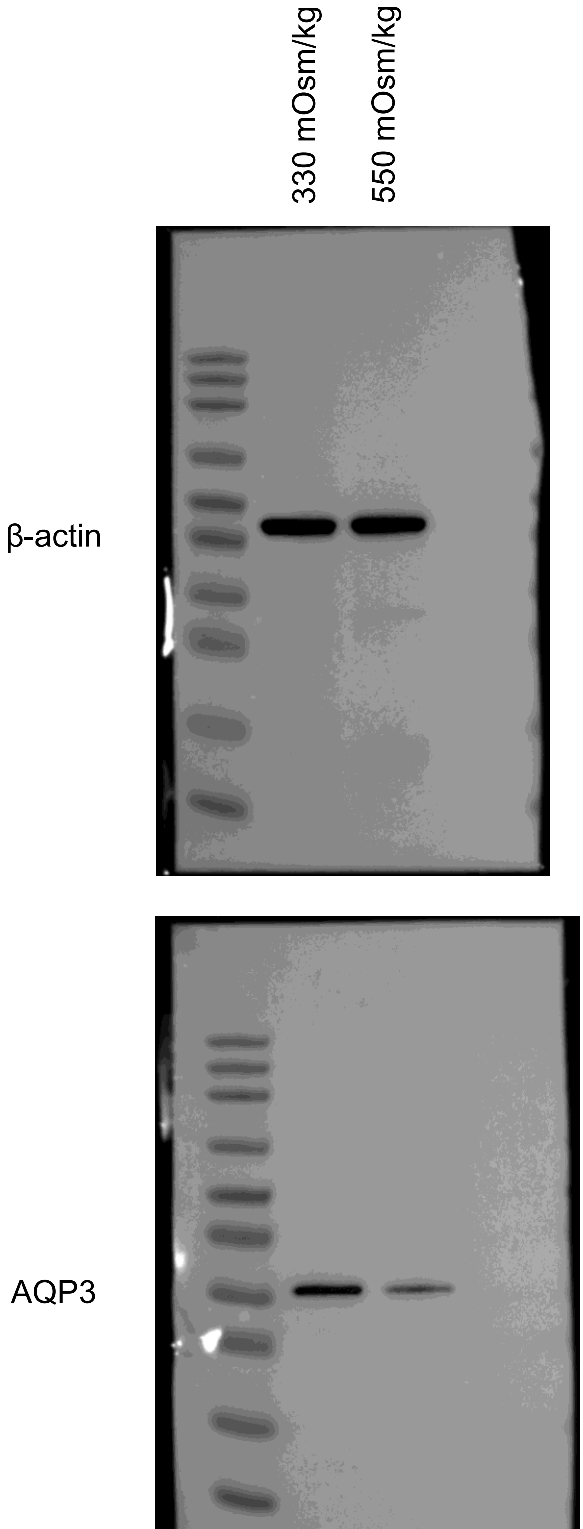
330 mOsm/kg
550 mOsm/kg
β-actin
AQP3

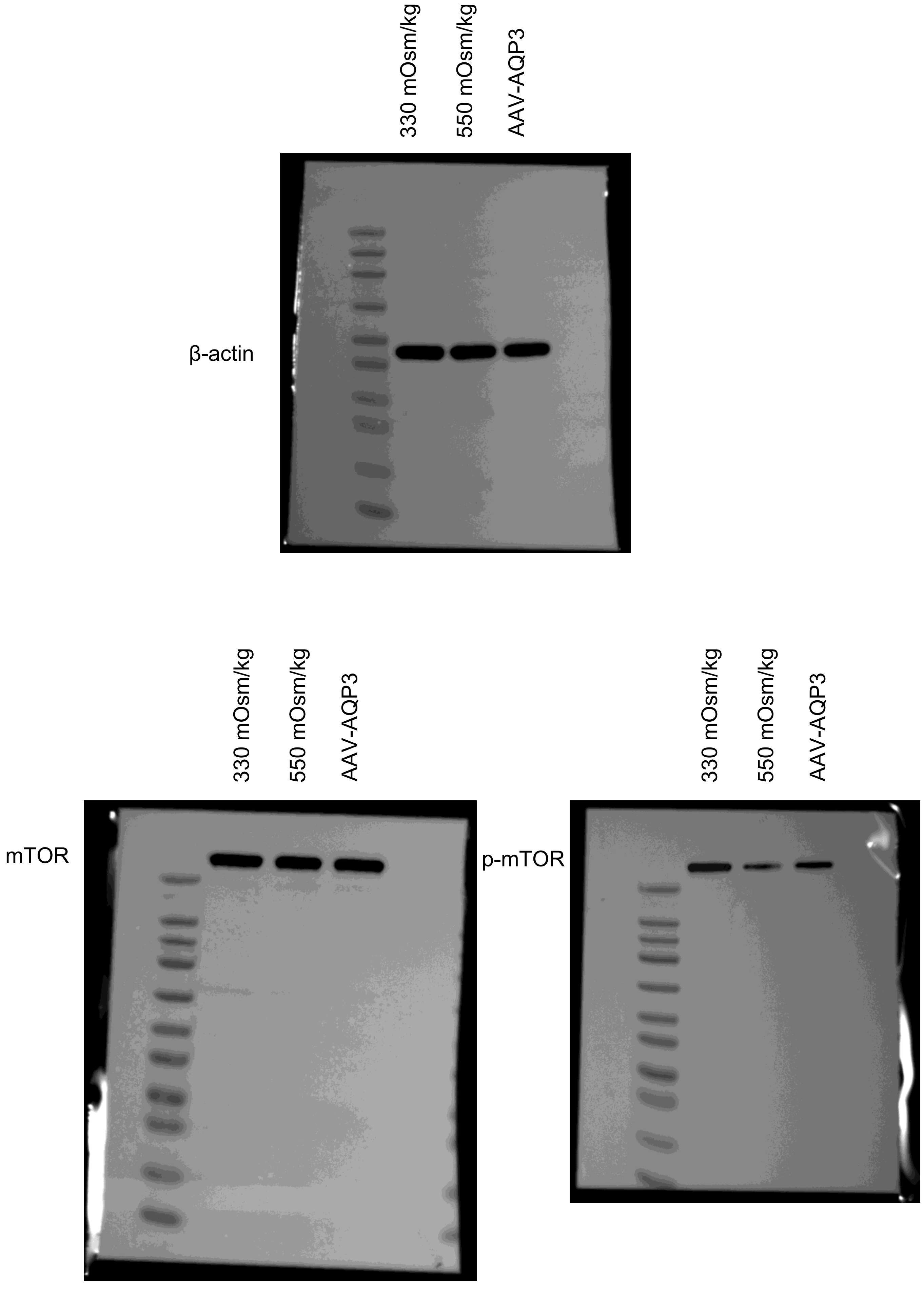
330 mOsm/kg
550 mOsm/kg
AAV-AQP3
β-actin
330 mOsm/kg
550 mOsm/kg
AAV-AQP3
mTOR
330 mOsm/kg
550 mOsm/kg
AAV-AQP3
p-mTOR

330 mOsm/kg
550 mOsm/kg
AAV-AQP3

PI3K

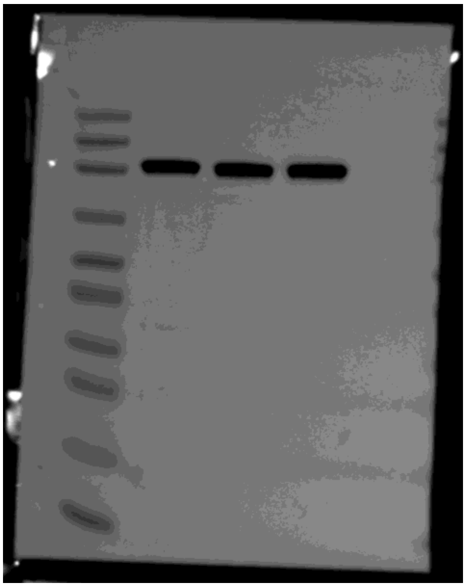

330 mOsm/kg
550 mOsm/kg
AAV-AQP3

p-PI3K

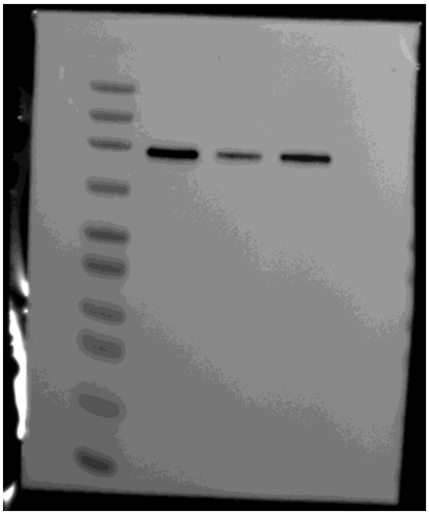

330 mOsm/kg
550 mOsm/kg
AAV-AQP3

Akt

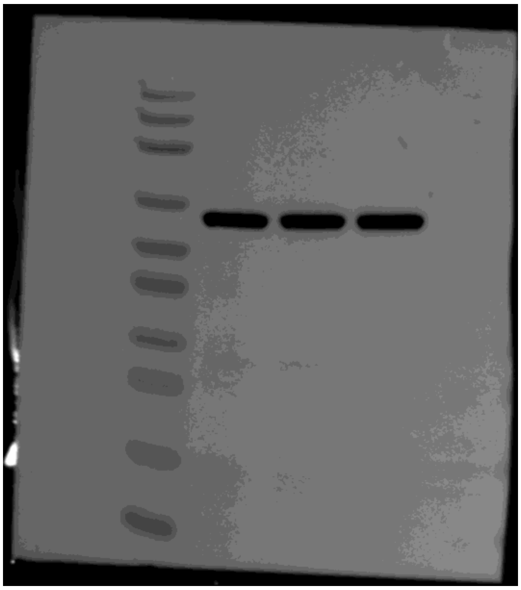

330 mOsm/kg
550 mOsm/kg
AAV-AQP3

p-Akt

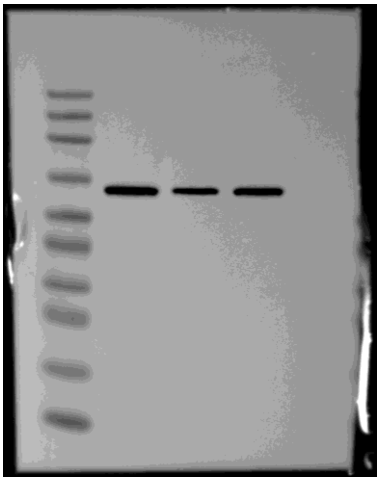

Supplement: Supplementary file 1 [file DataSheet1.pdf]
